# Sensitive UHPLC-MS/MS quantification method for 4β- and 4α-hydroxycholesterol in plasma for accurate CYP3A phenotyping

**DOI:** 10.1016/j.jlr.2022.100184

**Published:** 2022-02-16

**Authors:** Yosuke Suzuki, Ayako Oda, Jun Negami, Daiki Toyama, Ryota Tanaka, Hiroyuki Ono, Tadasuke Ando, Toshitaka Shin, Hiromitsu Mimata, Hiroki Itoh, Keiko Ohno

**Affiliations:** 1 Department of Medication Use Analysis and Clinical Research, Meiji Pharmaceutical University, Kiyose, Tokyo, Japan; 2 Department of Clinical Pharmacy, Oita University Hospital, Yufu, Oita, Japan; 3 Department of Urology, Oita University Faculty of Medicine, Yufu, Oita, Japan

**Keywords:** cholesterol, cytochrome P450, kidney, kinetics, pharmacokinetics, 4β-hydroxycholesterol, 4α-hydroxycholesterol, cytochrome P450 3A, mass spectrometry, plasma, 4α-OHC, 4α-hydroxycholesterol, 4β-OHC, 4β-hydroxycholesterol, APCI, atmospheric pressure chemical ionization, APPI, atmospheric pressure photoionization, CKD, chronic kidney disease, CV, coefficient of variation, CYP, cytochrome P450, HSA, human serum albumin, LC-MS/MS, high-performance liquid chromatography coupled to tandem mass spectrometry, LLOQ, lower limit of quantification, QC, quality control, UHPLC-MS/MS, ultra-high performance liquid chromatography coupled to tandem mass spectrometry

## Abstract

4β-Hydroxycholesterol (4β-OHC) is formed by Cytochrome P450 (CYP)3A and has drawn attention as an endogenous phenotyping probe for CYP3A activity. However, 4β-OHC is also increased by cholesterol autooxidation occurring in vitro due to dysregulated storage and in vivo by oxidative stress or inflammation, independent of CYP3A activity. 4α-hydroxycholesterol (4α-OHC), a stereoisomer of 4β-OHC, is also formed via autooxidation of cholesterol, not by CYP3A, and thus may have clinical potential in reflecting the state of cholesterol autooxidation. In this study, we establish a sensitive method for simultaneous quantification of 4β-OHC and 4α-OHC in human plasma using ultra-high performance liquid chromatography coupled to tandem mass spectrometry. Plasma samples were prepared by saponification, two-step liquid-liquid extraction, and derivatization using picolinic acid. Intense [M+H]+ signals for 4β-OHC and 4α-OHC di-picolinyl esters were monitored using electrospray ionization. The assay fulfilled the requirements of the US Food and Drug Administration guidance for bioanalytical method validation, with a lower limit of quantification of 0.5 ng/ml for both 4β-OHC and 4α-OHC. Apparent recovery rates from human plasma ranged from 88.2% to 101.5% for 4β-OHC, and 91.8% to 114.9% for 4α-OHC. Additionally, matrix effects varied between 86.2% and 117.6% for 4β-OHC and between 89.5% and 116.9% for 4α-OHC. Plasma 4β-OHC and 4α-OHC concentrations in healthy volunteers, stage 3–5 chronic kidney disease (CKD) patients, and stage 5D CKD patients as measured by the validated assay were within the calibration ranges in all samples. We propose this novel quantification method may contribute to accurate evaluation of in vivo CYP3A activity.

Pharmacokinetics of drugs show large interindividual variability, and some drug-metabolizing enzymes and transporters are involved in the variability. Cytochrome P450 (CYP)3A is a major subfamily of metabolic enzymes involved in the metabolism of some drugs in the liver and small intestine (1). The main isoenzymes of this subfamily are CYP3A4 and CYP3A5. There is a large interindividual variability in CYP3A activity among patients, and the variability was reported to affect the clinical efficacy and the adverse reaction of CYP3A substrate drugs (2, 3). Thus, phenotyping of CYP3A activity is clinically important for more effective and safer treatment by CYP3A substrate drugs.

Midazolam has been reported to be useful and considered a standard probe for CYP3A phenotyping (4, 5). Although midazolam is commonly used in drug-drug interaction studies (6, 7, 8, 9), this drug has some limitations in clinical application. For example, multiple blood samplings are needed to calculate the clearance for phenotyping, which limits its use in infants and elderly people. Midazolam shows high protein binding especially to albumin (approximately 96%) (10), and the free fraction may increase in patients with lower albumin levels, resulting in apparently increased hepatic clearance. Thus, phenotyping using midazolam may not be suitable in some patients with liver disease such as cirrhosis or kidney failure.

To overcome these problems, 4β-hydroxycholesterol (4β-OHC) has drawn attention as an endogenous phenotyping probe for CYP3A activity. 4β-OHC is formed by CYP3A4 and CYP3A5 (11, 12) and has a long plasma half-life (approximately 17 days) (13). Since there is no circadian change in plasma 4β-OHC concentrations, one-point blood sampling is sufficient for CYP3A phenotyping. 4β-OHC is slowly metabolized by CYP7A1 (14), and CYP7A1 activity is not affected by kidney failure (15). Therefore, plasma 4β-OHC concentration is a suitable probe for CYP3A phenotyping in infants, elderly people, and patients with kidney failure or liver diseases including cirrhosis (16, 17, 18, 19, 20, 21).

Several quantification methods have been reported for the measurement of plasma 4β-OHC concentrations using gas chromatography coupled to mass spectrometry (11) and high-performance liquid chromatography coupled to tandem mass spectrometry (LC-MS/MS) (22, 23, 24, 25, 26). Recently, Hautajärvi *et al.* (27) reported an ultra-high performance liquid chromatography coupled to high resolution mass spectrometry method for quantification of plasma 4β-OHC and 4α-hydroxycholesterol (4α-OHC) concentrations. 4α-OHC, a stereoisomer of 4β-OHC, is formed via autooxidation of cholesterol, and not by CYP3A. Therefore, plasma 4α-OHC concentration reflects plasma sample stability, because plasma 4α-OHC concentration increases in uncontrolled storage condition (28). Furthermore, oxysterols including 4β-OHC and 4α-OHC have been reported to be elevated by cholesterol autoxidation due to oxidative stress or inflammation in the liver, regardless of CYP3A activity (29). Thus, simultaneous quantification of 4β-OHC and 4α-OHC is preferred for phenotyping of CYP3A activity using clinical plasma samples.

In this study, we established a sensitive method for simultaneous quantification of 4β-OHC and 4α-OHC in human plasma using ultra-high performance liquid chromatography coupled to tandem mass spectrometry (UHPLC-MS/MS). The method was applied to measure plasma 4β-OHC and 4α-OHC concentrations in healthy volunteers and patients with chronic kidney disease (CKD).

## Material and methods

### Chemicals

The standards of 4β-OHC and 4β-OHC-D_7_ (4β-hydroxycholesterol-25, 26, 26, 26, 27, 27, 27-D_7_, isotopically labelled internal standard of 4β-OHC) were purchased from Avanti Polar Lipids, Inc (Alabama) and 4α-OHC standard from Toronto Research Chemicals (Ontario, Canada). The standards of 7α-OHC, 7β-OHC, 22(R)-OHC, 22(S)-OHC, 24(S)-OHC, 25-OHC, and 27-OHC, isomers of 4β-OHC and 4α-OHC, were purchased from Sigma-Aldrich (St. Louis). Human serum albumin (HSA) was purchased from FUJIFILM Wako Pure Chemical Corporation (Osaka, Japan). Other reagents (2-methyl-6-nitrobenzoic anhydride, 4-dimethylamino-pyridine, and picolinic acid) were purchased from Tokyo Chemical Industry Co, Ltd (Tokyo, Japan). Other solvents (water, methanol, 2-propanol, acetonitrile, n-hexane, formic acid, pyridine, triethylamine, and 28% sodium methoxide methanol solution) were of the highest analytical quality and were purchased from FUJIFILM Wako Pure Chemical Corporation.

### Healthy volunteers

Nine healthy volunteers who fasted for at least 8 h before blood sampling were recruited in this study. Blood was collected in tubes containing EDTA anticoagulant, centrifuged, and plasma samples were frozen at −80°C until measurement. Total cholesterol level was measured by enzymatic assay. This study was approved by the Ethics Committee of Meiji Pharmaceutical University (approval number: 202001). Prior explanations were provided to all volunteers to inform the scientific purpose of the study, and all volunteers gave written informed consent for participation in this study. Plasma obtained from healthy volunteers was used as unadulterated plasma for preparation of quality control (QC) samples and for validation purposes.

### CKD patients

Blood samples were collected from 15 patients with stage 3–5 CKD and from 14 patients with stage 5D CKD undergoing hemodialysis in Oita University Hospital. The patients fasted for at least 8 h before blood sampling. CKD stages were classified by estimated glomerular filtration rate according to the evidence-based clinical practice guideline for CKD published by Japanese Society of Nephrology (https://link.springer.com/content/pdf/10.1007%2Fs10157-014-0949-2.pdf, accessed 16 July 2020). Blood samples collected in tubes containing EDTA anticoagulant were centrifuged, and plasma samples were frozen at −80°C until assay. Total cholesterol level was measured by enzymatic assay. This study was approved by the Ethics Committees of Oita University Hospital (approval number: 1815) and the Ethics Committee of Meiji Pharmaceutical University (approval number: 202016). Prior explanations were provided to all patients to inform the scientific purpose of the study, and all patients gave written informed consent for participation in this study.

### Stock and working solutions

Independent 4β-OHC and 4α-OHC stock solutions were made separately for the preparation of calibration and QC samples. 4β-OHC and 4α-OHC were weighed in volumetric flasks and dissolved in 2-propanol (5 mg/50 ml and 1 mg/50 ml, respectively). The internal standard 4β-OHC-D_7_ was also weighed in volumetric flasks and dissolved in 2-propanol (20 μg/ml). The stock solutions were stored at −80°C.

All calibrating and QC solutions contained a mixture of 4β-OHC and 4α-OHC. Eight different calibrating solutions containing equal concentrations of 4β-OHC and 4α-OHC at 10, 20, 40, 100, 400, 1000, 4000, and 10,000 ng/ml (final concentrations in calibration samples: 0.5, 1, 2, 5, 20, 50, 200, and 500 ng/ml) were prepared by diluting the respective calibrating stock solutions with 2-propanol. QC solutions were prepared at lower limit of quantification (LLOQ), low (QCA), medium (QCB), and high (QCC) concentrations containing equal concentrations of 4β-OHC and 4α-OHC at 10, 30, 500, and 8000 ng/ml (final concentrations in QC samples: 0.5, 1.5, 25, and 400 ng/ml), respectively, by diluting the respective QC stock solutions with 2-propanol.

### Derivatization solution

A derivatization solution was prepared by mixing the following reagents: 250 mg of 2-methyl-6-nitrobenzoic anhydride, 75 mg of 4-dimethylamino-pyridine, 200 mg of picolinic acid, 7.5 ml of pyridine, and 1 ml of triethylamine. The derivatization solution was freshly prepared just before plasma sample preparation.

### Plasma sample preparation

For measurement of 4β-OHC and 4α-OHC concentrations, plasma samples were pretreated as follows. In each 2.0-mL safe-lock tube, 50 μl of subject’s plasma sample was mixed with 50 μl of internal standards solution (20 ng/ml of 4β-OHC-D_7_) and 2.5 μl of 2-propanol for volume adjustment. After adding 200 μl of 28% sodium methoxide methanol solution, the mixture was vortex-mixed for 30 s and left at room temperature (15–25°C) for 20 min. Then, 250 μl of water and 1 ml of n-hexane were added, vortex-mixed for 5 min, and centrifuged (10 min at 1,500 *g*, 25°C). The supernatant (700 μl) was transferred to a clean 1.5-ml safe-lock tube and evaporated to dryness under a N_2_ gas stream at 40^°^C. The residue was reconstituted with 100 μl of derivatization solution, vortex-mixed for 30 s, and left at room temperature for 30 min. Then, 1 ml of n-hexane was added, vortex-mixed for 5 min, and centrifuged (5 min at 1,500 *g*, 25°C). The supernatant (700 μl) was transferred to a clean 1.5-ml safe-lock tube and evaporated to dryness under a N_2_ gas stream at 40°C. The residue was reconstituted with 100 μl of acetonitrile, vortex-mixed, and transferred to an autosampler vial.

QC sample was prepared by spiking 50 μl of unadulterated plasma with 2.5 μl of QC solution containing 4β-OHC and 4α-OHC. Calibration sample was prepared by spiking 50 μl of 2% HSA solution (blank matrix) with 2.5 μl of calibration solution containing 4β-OHC and 4α-OHC. Each calibration or QC sample was mixed with 50 μl of internal standards solution. Then, these samples were processed as for subjects’ plasma samples.

### Instrumental analysis parameters

The UHPLC-MS/MS system (Shimadzu, Kyoto, Japan) consisted of a Nexera X2 LC system and a quadrupole mass spectrometer (LCMS-8040). For chromatographic separation, a Waters Acquity BEH C_18_ column (1.7 μm, 2.1 × 150 mm) and a Waters Acquity BEH C_18_ VanGuard precolumn (1.7 μm, 2.1 × 5 mm) were used at 55°C. 0.1% aqueous formic acid (A) and acetonitrile with 0.1% formic acid (B) were used as mobile phase. The gradient started at 80% B (0.5 min). The ratio was changed linearly to 95% B within 5.5 min and maintained for another 4 min. Then, the ratio was returned to 80% B and maintained for another 2.5 min. The flow rate was 0.4 ml/min, and the injection volume was 2 μl.

The ionization parameters were as follows: desolvation line temperature 250°C, heat block temperature 400°C, nebulizer gas flow 3 L/min, drying gas flow 15 L/min, and collision induced dissociation gas pressure 230 kPa. The mass spectrometer was tuned automatically to 4β-OHC and 4α-OHC and the internal standard using LabSolutions LCMS software (Shimadzu). Multiple reaction monitoring analysis was performed using argon as collision induced dissociation gas. The MS/MS transitions for the di-picolinyl esters and the electrode voltage of Q1 prebias, collision cell Q2, and Q3 prebias were as follows: m/z 613.3→m/z 490.5, −24 V, −13 V, and −16 V, respectively, for 4β-OHC and 4α-OHC and m/z 620.3→m/z 497.6, −24 V, −15 V, and −16 V for 4β-OHC-D_7_ (supplemental Fig. S1). The dwell time for each transition was 0.1 s.

### Full validation of the analytical method

Analytical full validation was conducted according to the recommendations published by the US Food and Drug Administration (https://www.fda.gov/media/70858/download, accessed 21 April 2020) as described in our previous studies (30, 31). Each validation batch contained eight calibration samples and 30 QC samples at different concentrations [blank (endogenous level), LLOQ, QCA, QCB, and QCC in plasma; in sextuplicate each], and three validation batches were analyzed. Accuracy (%), a percentage of the measured concentration to the nominal concentration, and precision [% coefficient of variation (CV)], a percentage of the observed SD to the mean measured concentration, were calculated for individual analytical batch (within-batch) and for three validation batches (batch-to-batch). Extraction apparent recovery rates from plasma were calculated by comparing the peak areas from QCA, QCB, and QCC obtained from validation analysis with the respective peak areas obtained from unadulterated plasma (from 6 different healthy volunteers) spiked at these QC levels after extraction (representing 100% of the analyte amount in an identical matrix), in sextuplicate determination. Matrix effects were evaluated by comparing peak areas of unadulterated plasma samples (from 6 different healthy volunteers) spiked at QC levels A-C after extraction with the respective peak areas of matrix-free LC eluent containing 100% of standards and internal standards, in sextuplicate determination. Stability of the analytes was evaluated using QCB and QCC samples subjected to three freeze-and-thaw cycles, and the respective accuracies were calculated. Stability in the autosampler was evaluated by measuring QCB and QCC samples after standing in the autosampler at 10°C for 24 h, and the respective accuracies were calculated. Long-term stability of 4β-OHC and 4α-OHC has been reported (22).

### Stability of 4β-OHC and 4α-OHC over time in plasma under various conditions

Changes in plasma 4β-OHC and 4α-OHC concentrations over time were assessed under various conditions in vitro. Fresh unadulterated plasma samples from six healthy volunteers were dispensed in open or safe-lock tubes and stored at room temperature or 4°C for 1, 2, 7, 14, and 30 days, each tested in sextuplicate. After storage, 4β-OHC and 4α-OHC concentrations were determined by the UHPLC-MS/MS method developed. Measured concentrations of 4β-OHC and 4α-OHC in each sample were compared with the concentrations in freshly prepared samples without storage and expressed as percentage. 4β-OHC and 4α-OHC were considered stable when the residual percentage was within 85%–115%.

### Data analysis and statistics

Calibration curves for 4β-OHC and 4α-OHC were constructed using the calibration samples and analyte-specific multiple reaction monitoring quantifier transitions. Peak area ratios of each analyte to internal standard were calculated, and weighted linear regression (1/x) was performed for each analytical batch using the LabSolutions LCMS software (Shimadzu). Data are expressed as mean ± SD. Differences between healthy volunteers, stage 3–5 CKD patients, and stage 5D CKD patients were analyzed using one-way ANOVA with Dunnett’s posthoc test or Kruskal-Wallis test with Dunn's posthoc test. A *P* value less than 0.05 was considered statistically significant. Statistical analyses were performed using Graph Pad Prism 7 (GraphPad Software, La Jolla, CA).

## Results

### Mass spectrometric and chromatographic characteristics

Intense [M+H]^+^ signals for 4β-OHC and 4α-OHC di-picolinyl esters were monitored using ESI. The same mass transition (m/z 613.3→m/z 490.5) was monitored for 4β-OHC and 4α-OHC because they are the stereoisomers. The mass transition of internal standard 4β-OHC-D_7_ (m/z 620.3→m/z 497.6) differed from that of the two analytes by only the mass shift due to isotopic labeling.

**Fig. 1:**
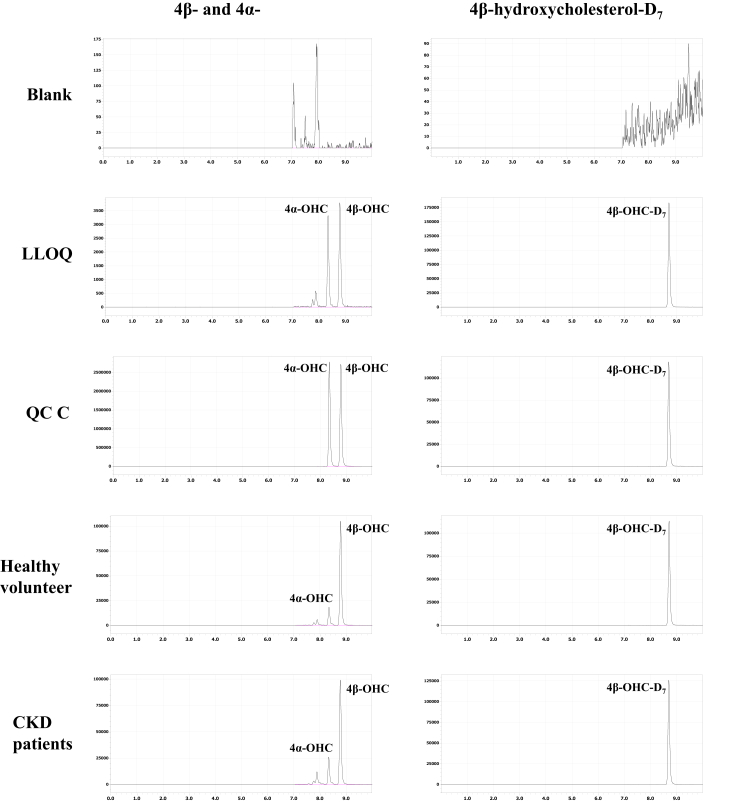
Selected ultra-high performance liquid chromatography with tandem mass spectrometry (UHPLC-MS/MS) chromatograms of 4β-OHC, 4α-OHC, and 4β-OHC-D_7_ in plasma samples. From top down: blank matrix (2% human serum albumin solution), LLOQ sample in blank matrix (0.5 ng/ml), QCC in plasma (400 ng/ml), plasma sample of healthy volunteer (calculated 4β-OHC and 4α-OHC concentrations: 27.3 and 4.5 ng/ml), and plasma sample of CKD patient (calculated 4β-OHC and 4α-OHC concentrations: 22.9 and 4.9 ng/ml). 4α-OHC, 4α-hydroxycholesterol; 4β-OHC, 4β-hydroxycholesterol; CKD, chronic kidney disease; LLOQ, lower limit of quantitation; QC, quality control.

Figure 1 shows the chromatograms of 4β-OHC, 4α-OHC, and 4β-OHC-D_7_ for 2% HSA solution (surrogate matrix), LLOQ sample, QCC sample, plasma sample of a healthy volunteer, and plasma sample of a CKD patient. Retention times were approximately 8.3 and 8.8 min after injection for 4β-OHC and 4α-OHC, respectively. In the chromatogram, 4β-OHC and 4α-OHC were separated from the isomers comprising 7α-OHC, 7β-OHC, 22(R)-OHC, 22(S)-OHC, 24(S)-OHC, 25-OHC, and 27-OHC (supplemental Fig. S2).

### Validation results

For 4β-OHC, the correlation coefficient (*r*^2^) of the calibration curve for the calibration range of 0.5–500 ng/ml was ≥0.9986. For 4α-OHC, *r*^2^ for the same calibration range was 0.9989.

Within-batch and batch-to-bath accuracy and precision for 4β-OHC are shown in Table 1. Within-batch accuracy and precision for QCA, QCB, and QCC ranged from 98.6% to 103.1% and 1.3% to 4.7% CV, respectively. Batch-to-batch accuracy and precision for the three QCs ranged from 100.2% to 101.7% and 3.6% to 4.8% CV, respectively. Within-batch accuracy and precision for LLOQ ranged from 96.5% to 102.3% and 2.1% to 4.0% CV, and batch-to-batch accuracy and precision were 99.4% and 3.9% CV, respectively. Within-batch and batch-to-bath accuracy and precision for 4α-OHC are shown in Table 2. Within-batch accuracy and precision for QCA, QCB, and QCC ranged from 94.2% to 106.0% and 2.5% to 7.8% CV, respectively. Batch-to-batch accuracy and precision for the three QCs ranged from 95.4% to 101.8% and 4.7% to 7.7% CV, respectively. Within-batch accuracy and precision for LLOQ ranged from 95.5% to 103.8% and 1.2% to 3.9% CV, and batch-to-batch accuracy and precision were 101.5% and 6.3% CV, respectively.

**Table 1:** Summary of validation results for 4β-OHC concentrations in human plasma

		Nominal 4β-OHC Concentrations (ng/ml)				
		Endogenous	LLOQ	QCA	QCB	QCC
		-	0.5 + Endo	1.5 + Endo	25 + Endo	400 + Endo
Within-batch						
1	Mean (ng/ml)	40.1	39.4	42.9	67.0	441.7
	Accuracy (%)	-	96.9	103.2	102.9	100.3
	Precision (%CV)	9.3	2.3	3.6	5.8	4.7
2	Mean (ng/ml)	40.7	40.8	42.5	70.0	462.0
	Accuracy (%)	-	99.0	100.8	106.6	104.8
	Precision (%CV)	3.7	3.4	3.5	6.5	6.1
3	Mean (ng/ml)	37.1	37.3	39.2	61.9	402.4
	Accuracy (%)	-	99.2	101.7	99.7	92.1
	Precision (%CV)	5.4	1.8	1.9	6.7	6.4
Batch-to-batch						
Accuracy (%)		-	98.3	101.9	103.1	98.5
Precision (%CV)		7.5	4.5	5.1	8.0	8.3
Apparent recovery rate [%, mean (range)]		-	-	92.0 (89.4–93.7)	93.6 (88.2–100.2)	98.0 (94.9–101.5)
Matrix effect [%, mean (range)]		-	-	108.9 (103.9–112.5)	99.4 (86.2–117.6)	102.0 (96.7–107.7)

4β-OHC, 4β-hydroxycholesterol; CV, coefficient of variation; Endo, endogenous level; LLOQ, lower limit of quantitation; QC, quality control; QCA, low; QCB, medium; QCC, high.

**Table 2:** Summary of validation results for 4α-OHC concentrations in human plasma

		Nominal 4α-OHC Concentrations (ng/ml)				
		Endogenous	LLOQ	QCA	QCB	QCC
		-	0.5 + Endo	1.5 + Endo	25 + Endo	400 + Endo
Within-batch						
1	Mean (ng/ml)	6.9	6.9	8.9	33.8	409.1
	Accuracy (%)	-	93.4	105.7	106.0	100.5
	Precision (%CV)	5.9	2.9	2.9	6.4	6.3
2	Mean (ng/ml)	6.8	6.9	8.6	34.1	387.3
	Accuracy (%)	-	95.1	103.6	107.2	95.2
	Precision (%CV)	7.7	6.7	4.0	5.2	5.1
3	Mean (ng/ml)	6.9	7.2	8.2	31.1	365.9
	Accuracy (%)	-	97.5	96.9	97.3	89.9
	Precision (%CV)	10.4	5.6	3.2	9.9	4.8
Batch-to-batch						
Accuracy (%)		-	95.5	101.6	103.2	95.2
Precision (%CV)		7.8	5.6	4.7	8.1	6.7
Apparent recovery rate [%, mean (range)]		-	-	101.1 (97.7–106.9)	103.6 (91.8–112.3)	105.4 (92.8–114.9)
Matrix effect [%, mean (range)]		-	-	101.5 (97.2–108.2)	100.4 (89.5–116.9)	101.8 (90.2–113.5)

4α-OHC, 4α- hydroxycholesterol; CV, coefficient of variation; Endo, endogenous level; LLOQ, lower limit of quantitation; QC, quality control; QCA, low; QCB, medium; QCC, high.

The apparent recovery rates and matrix effects of 4β-OHC for QCA, QCB, and QCC ranged from 88.2% to 101.5% and 86.2% to 117.6%, respectively (Table 1). The apparent recovery rates and matrix effects of 4α-OHC for QCA, QCB, and QCC ranged from 91.8% to 114.9% and 89.5% to 116.9%, respectively (Table 2).

The three-cycle freeze-and-thaw stability test for QCB and QCC revealed no significant changes in measured concentrations, with accuracy ranging from 99.4% to 104.2% for 4β-OHC and 103.4% to 112.5% for 4α-OHC. Stability test in the autosampler was also acceptable for QCB and QCC, with accuracy ranging from 100.7% to 104.3% for 4β-OHC and 94.0% to 105.3% for 4α-OHC.

### Changes in 4β-OHC and 4α-OHC concentrations in human plasma under various conditions

**Fig. 2:**
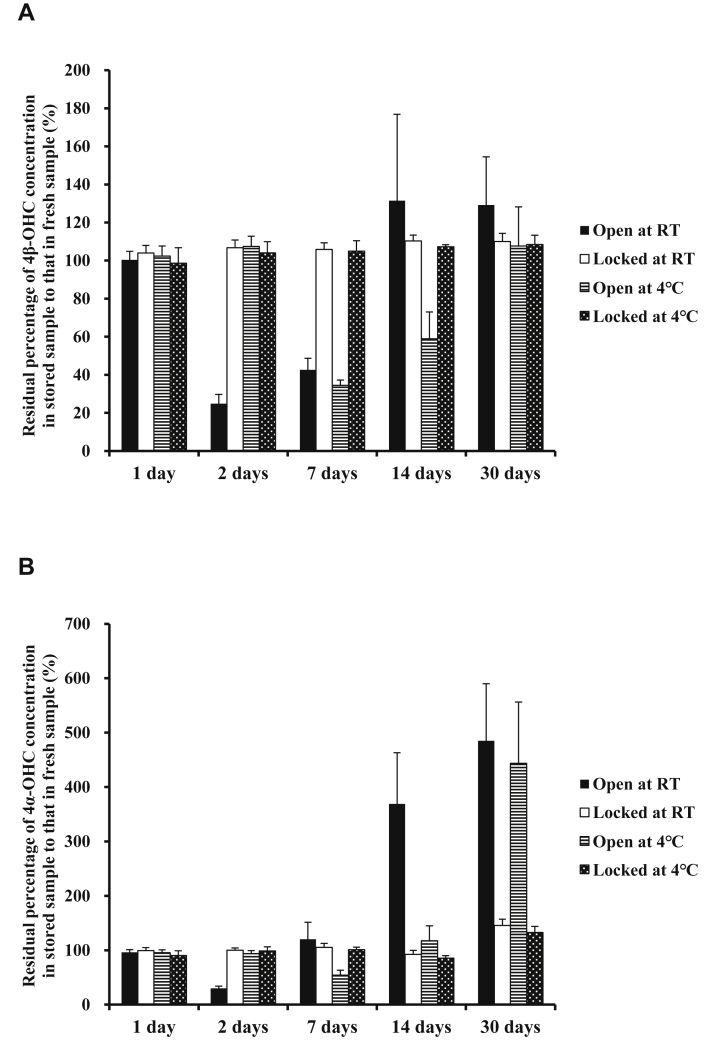
Changes 4β-OHC (A) and 4α-OHC (B) concentrations over time in unadulterated plasma samples from six healthy volunteers stored under various conditions for 30 days. 4α-OHC, 4α-hydroxycholesterol; 4β-OHC, 4β-hydroxycholesterol.

Figure 2 shows the changes in plasma 4β-OHC and 4α-OHC concentrations in plasma samples stored in various conditions. Plasma 4β-OHC concentration was stable for 30 days in safe-lock tubes stored at room temperature and 4°C. On the other hand, plasma 4β-OHC concentration decreased on day 2 followed by an increase from day 7 in open tubes at room temperature and decreased on day 7 followed by an increase from day 14 in open tubes at 4°C. Similarly, when stored in open tubes, plasma 4α-OHC concentration decreased on day 2 followed by a sharp increase from day 7 at room temperature and decreased on day 7 followed by a sharp increase from day 14 at 4°C. Plasma 4α-OHC concentration was stable for 14 days in safe-lock tubes at room temperature and 4°C, followed by an increase on day 30.

### Application of the method to measure 4β-OHC and 4α-OHC in healthy volunteers and CKD patients

**Fig. 3:**
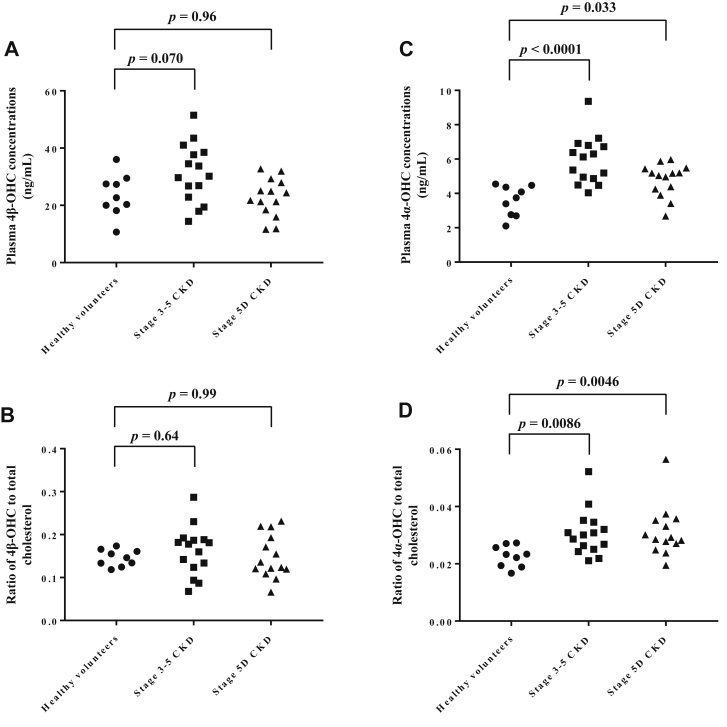
Plasma 4β-OHC concentrations (A), ratios of 4β-OHC to total cholesterol (B), plasma 4α-OHC concentrations (C), and ratios of 4α-OHC to total cholesterol in healthy volunteers, stage 3–5 CKD patients, and stage 5D CKD patients. Data were analyzed by one-way ANOVA with Dunnett’s post hoc test (A–C) or Kruskal-Wallis test with Dunn's post hoc test (D). 4α-OHC, 4α-hydroxycholesterol; 4β-OHC, 4β-hydroxycholesterol; CKD, chronic kidney disease.

The validated UHPLC-MS/MS method was used to simultaneously measure plasma concentrations of 4β-OHC and 4α-OHC in healthy volunteers and CKD patients (Table 3). Serum creatinine was elevated in stage 3–5 CKD patients and stage 5D CKD patients. As shown in Fig. 3A, plasma 4β-OHC concentrations were 23.6 ± 7.4 in healthy volunteers, 31.2 ± 10.3 in stage 3–5 CKD patients, and 22.7 ± 6.7 ng/ml in stage 5D CKD patients, with a significant difference among three groups by one-way ANOVA (*P* = 0.022), whereas no significant difference was detected by posthoc test. There was no significant difference in the ratio of 4β-OHC to total cholesterol among three groups (*P* = 0.65) (Fig. 3B).

**Table 3:** Clinical characteristics of healthy volunteers and CKD patients

Characteristic	Healthy Volunteers	Stage 3–5 CKD Patients	Stage 5D CKD Patients
Number	9	15	14
Males/females	7/2	13/2	12/2
Age (year)	25.0 ± 4.4 [21–35]	44.9 ± 13.0 [27–66]	48.1 ± 13.8 [31–73]
Body weight (kg)	60.7 ± 8.6 [45.0–74.5]	66.2 ± 15.7 [39.5–90.6]	64.3 ± 13.3 [38.9–90.0]
ALT (IU/L)	16.0 ± 4.9 [9.0–23.0]	17.6 ± 11.0 [5.5–46.2]	7.7 ± 4.0 [3.8–16.4]
Total bilirubin (mg/dl)	0.67 ± 0.30 [0.30–1.30]	0.55 ± 0.23 [0.29–1.21]	0.47 ± 0.12 [0.27–0.72]
Serum creatinine (mg/dl)	0.78 ± 0.20 [0.53–1.00]	1.67 ± 0.36 [1.05–2.37]	10.4 ± 3.0 [6.0–17.2]
Total cholesterol (mg/dl)	159.4 ± 38.9 [90–224]	195.6 ± 23.4 [150.0–226.2]	160.0 ± 39.6 [92.0–202.9]

ALT, alanine aminotransaminase; CKD, chronic kidney disease.

Data are expressed as numbers or mean ± SD [range].

Plasma 4α-OHC concentrations were 3.6 ± 0.9 in healthy volunteers, 5.9 ± 1.4 in stage 3–5 CKD patients, and 4.8 ± 0.9 ng/ml in stage 5D CKD patients; a significant difference was observed among three groups by one-way ANOVA (*P* < 0.0001), and significant differences were also detected by posthoc test (Fig. 3C). Similarly, a significant difference in the ratio of 4α-OHC to total cholesterol was observed among three groups by Kruskal-Wallis test (*P* = 0.0045), and significant differences were also detected by posthoc test (Fig. 3D).

**Fig. 4:**
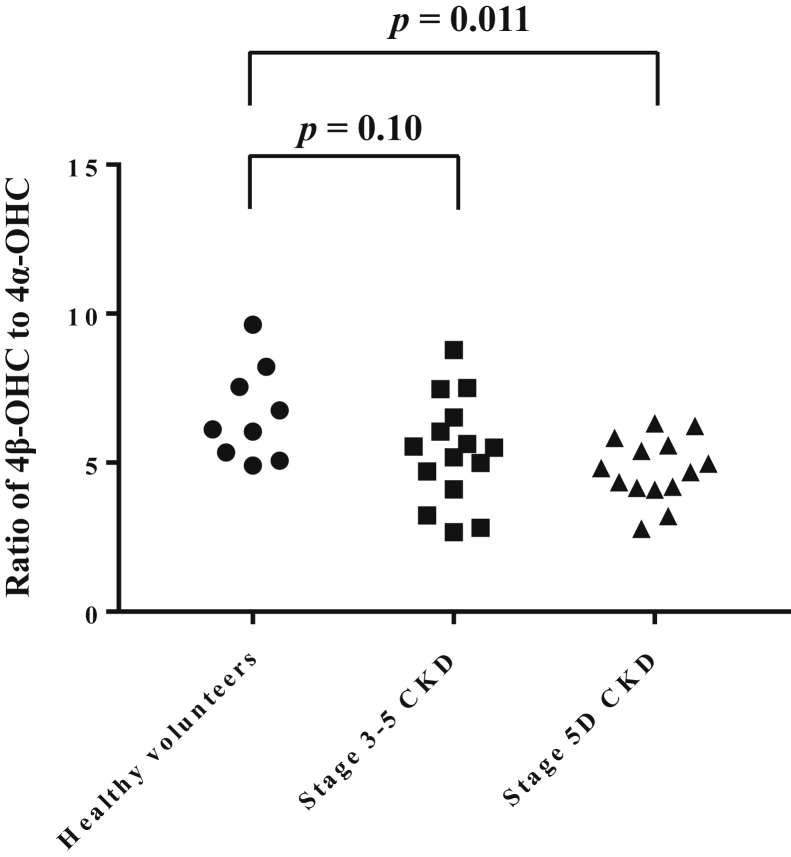
Ratio of 4β-OHC to 4α-OHC concentration in healthy volunteers, stage 3–5 CKD patients, and stage 5D CKD patients. Data were analyzed by one-way ANOVA with Dunnett’s post hoc test. 4α-OHC, 4α-hydroxycholesterol, 4β-OHC, 4β-hydroxycholesterol; CKD, chronic kidney disease.

The ratios of 4β-OHC to 4α-OHC were 6.6 ± 1.6 in healthy volunteers, 5.4 ± 1.8 in stage 3–5 CKD patients, and 4.8 ± 1.1 in stage 5D CKD patients, with a significant difference among three groups by one-way ANOVA (*P* = 0.021) (Fig. 4). Posthoc test revealed a significantly smaller 4β-OHC to 4α-OHC ratio in stage 5D CKD patients compared to healthy volunteers. All measured concentrations of 4β-OHC and 4α-OHC were within the calibration ranges.

## Discussion

In 2001, Bodin *et al.* (11) reported for the first time that plasma 4β-OHC concentration was useful for phenotyping of CYP3A activity in vivo. Since then, many studies have evaluated the usefulness of plasma 4β-OHC concentration as an endogenous marker for phenotyping CYP3A activity. Interestingly, these studies reported the pros and cons of the use of plasma 4β-OHC concentration. For example, Gravel *et al.* (32) recently reported that 4β-OHC was a valid and convenient marker for phenotyping CYP3A activity with high correlation with midazolam pharmacokinetics. On the other hand, a recent review by Penzak *et al.* (33) did not support the use of 4β-OHC as an endogenous biomarker for phenotyping CYP3A activity, because of the mild correlation with midazolam pharmacokinetics. The inconsistency may be partially explained by autooxidation of cholesterol. 4β-OHC is produced by in vivo hydroxylation of cholesterol at the 4β-position by CYP3A, followed by in vitro autooxidation of cholesterol after blood sampling (28). Thus, improper control of blood samples, such as prolonged storage at room temperature in open tubes, may increase plasma 4β-OHC concentration after blood sampling, causing inaccurate CYP3A phenotyping. Furthermore, 4β-OHC was reported to be produced in vivo by autooxidation of cholesterol due to oxidative stress or inflammation in the liver (29). Like 4β-OHC, 4α-OHC is produced by autooxidation of cholesterol, but 4α-OHC is not formed by CYP3A in vivo (28). Therefore, plasma 4α-OHC concentration has clinical potential in providing information on the status of cholesterol autooxidation, i.e., plasma 4β-OHC concentration may not accurately indicate CYP3A activity if plasma 4α-OHC concentration is high. Thus, simultaneous quantification of 4β-OHC and 4α-OHC may enhance the accuracy of 4β-OHC for phenotyping CYP3A.

Three studies have already reported methods of simultaneous measurement of plasma 4β-OHC and 4α-OHC concentrations. Bodin *et al.* (11) reported a simultaneous quantification method using gas chromatography coupled to mass spectrometry, but the method was not validated. Goodenough *et al.* (22) reported an LC-MS/MS method, which was validated for 4β-OHC but not for 4α-OHC. Recently, Hautajärvi *et al.* (27) reported a validated method for simultaneous quantification of 4β-OHC and 4α-OHC in plasma using ultra-high performance liquid chromatography coupled to high resolution mass spectrometry. Their method allowed accurate measurements of 4β-OHC and 4α-OHC, with LLOQ of 0.5 and 2 ng/ml for 4β-OHC and 4α-OHC, respectively, using 100 μl of plasma sample. However, the LLOQ for 4α-OHC may not be low enough, because 4α-OHC concentrations in human plasma were reported to be 2.12–5.65 ng/ml in 24 subjects (27). The method of Hautajärvi *et al.* avoided the derivatization process in plasma sample preparation and monitored sodium adducted ions, causing inadequate sensitivity. Our novel method adopted a short derivatization process using picolinic acid and monitored the proton adducted ions, which increased ionization efficiency and achieved better LLOQ of 0.5 ng/ml for 4α-OHC. Our newly developed method thus has the advantage of having sufficient sensitivity to evaluate plasma 4α-OHC concentrations in different clinical settings, and it seems to be superior to previous reported methods for simultaneous quantification of 4β-OHC and 4α-OHC in human plasma.

We prepared plasma samples by saponification using sodium methoxide, two-step liquid-liquid extraction using n-hexane, and derivatization using picolinic acid. Sodium methoxide was used for saponification of 4β-OHC and 4α-OHC comprised in lipoproteins and was utilized in previous reports (23, 26, 27). Derivatization is an option to enhance ionization efficiency because 4β-OHC and 4α-OHC has poor ionization efficiency, but the process is not essential for LC-MS/MS method. Van de Merbel *et al.* (23) and Hasan *et al.* (26) quantified 4β-OHC without derivatization using LC-MS/MS equipped with atmospheric pressure chemical ionization (APCI) and atmospheric pressure photoionization (APPI) probes, respectively. APCI and APPI provide efficient ionization for relatively nonpolar compounds such as steroids and are suggested to be feasible methods for quantification of 4β-OHC and 4α-OHC. However, APCI and APPI seemed to be inferior to ESI with derivatization process in sensitivity for 4β-OHC (23, 26). Thus, APCI and APPI may not be suitable for sensitive quantification, especially for 4α-OHC, and ESI with derivatization using picolinic acid achieves sensitive quantification of 4β-OHC and 4α-OHC in plasma.

Validation results for 4β-OHC and 4α-OHC are within the acceptable ranges according to the recommendations published by the US Food and Drug Administration. High apparent recovery rates and matrix effects suggest effective sample preparation and minimal ion suppression or enhancement. When samples exposed to air were stored under various in vitro conditions, plasma 4β-OHC and 4α-OHC concentrations increased after a transient decrease. However, 4β-OHC and 4α-OHC were stable when stored in safe-lock tubes, suggesting increased stability by avoiding contact with air. The elevation is speculated to be due to autooxidation of cholesterol. However, detailed mechanism of the transient decrease in plasma 4β-OHC and 4α-OHC concentrations is unknown, but other pathways such as additional autooxidation of 4β-OHC and 4α-OHC may be involved. These findings suggest that plasma samples should be stored in rocked tubes to avoid autooxidation of cholesterol to 4β-OHC and 4α-OHC.

The validated UHPLC-MS/MS method was used to measure plasma 4β-OHC and 4α-OHC concentrations in healthy volunteers and CKD patients. Plasma 4β-OHC concentrations in this study were similar to previously reported values in healthy volunteers (11, 22, 23, 27), stage 3–5 CKD patients (18, 21), and stage 5D CKD patients (17, 19). Plasma 4α-OHC concentrations in healthy volunteers in this study were also similar to previous reports (11, 22, 27). To the best of our knowledge, this is the first report of plasma 4α-OHC concentrations in CKD patients. All the measured concentrations were within the calibration ranges. Especially, our novel method with lower LLOQ for 4α-OHC achieved to measure plasma 4α-OHC concentrations well in advance in healthy volunteers. These suggest that the established method is clinically useful for measuring 4β-OHC and 4α-OHC concentrations in human plasma samples from healthy volunteers as well as CKD patients and would contribute to accurate evaluation of in vivo CYP3A activity.

The 4β-OHC to total cholesterol ratio has been reported to be superior to 4β-OHC alone for CYP3A phenotyping (34). However, there was no significant difference in 4β-OHC to total cholesterol ratio among three groups (Fig. 3B), although CKD was reported to decrease CYP3A activity in patients (35). On the other hand, a significant difference in 4α-OHC to total cholesterol ratio was observed among three groups, with significant increases in stage 3–5 CKD patients (0.030 ± 0.0079) and stage 5D CKD patients (0.031 ± 0.0088) compared to healthy volunteers (0.023 ± 0.0037) (Fig. 3D). Studies have shown that increases in 4β-OHC and 4α-OHC by cholesterol autoxidation were caused not only by in vitro uncontrolled storage condition but also by in vivo oxidative stress or inflammation (28, 29). Moreover, elevated oxidative stress and inflammation are observed in CKD patients (36, 37). In this study, collection, storage, and pretreatment of blood samples from healthy volunteers, stage 3–5 CKD patients, and stage 5D CKD patients were conducted under the same conditions; therefore, the degree of in vitro cholesterol autooxidation of all samples probably did not vary greatly. Therefore, the significant elevations of 4α-OHC concentration and 4α-OHC to cholesterol ratio in stage 3–5 CKD and stage 5D CKD patients compared to healthy subjects likely reflect the elevated oxidative stress and inflammation in these patients. Similarly, elevated oxidative stress and inflammation in stage 3–5 CKD patients and stage 5D CKD patients are probably involved in the elevation of 4β-OHC concentration in these patients and in apparently no change in plasma 4β-OHC concentration among three groups. Thus, correction by plasma 4α-OHC concentration may control the fluctuation of in vitro and in vivo cholesterol autoxidation among patients and improve the accuracy of 4β-OHC as an endogenous biomarker for CYP3A phenotyping. Indeed, as shown in Fig. 4, a significant difference in 4β-OHC to 4α-OHC ratio was observed among three groups, with a significant difference between healthy volunteers and stage 5D CKD patients. To the best of our knowledge, this is the first report of the potential use of the 4β-OHC to 4α-OHC ratio in CYP3A phenotyping. Further large-scale study is needed to validate this finding.

Our method has some limitations. First, because the retention times of 4α-OHC and 4β-OHC-D_7_ were different, the matrix effect of the 4α-OHC assay was not corrected by internal standard. However, the variation due to matrix effect was not large in the 4α-OHC assay and thus appeared to have little effect on the results. Second, the rationale for the use of 4β-OHC to 4α-OHC ratio in CYP3A phenotyping is not well established. We showed the potential of 4β-OHC to 4α-OHC ratio for cross-sectional CYP3A phenotyping in healthy volunteers and CKD patients in this study, but the interindividual variability in 4β-OHC to 4α-OHC ratio was large within each group, although a significant difference was observed among three groups (Fig. 4). To establish a better clinical approach for CYP3A phenotyping, further clinical study by approaches such as model-based analysis using 4β-OHC and 4α-OHC concentrations is needed.

In conclusion, simultaneous quantification method for plasma 4β-OHC and 4α-OHC concentrations using UHPLC-MS/MS was developed and validated and was applied to measurement of plasma concentrations in healthy volunteers and CKD patients. Our novel method may contribute to accurate evaluation of in vivo CYP3A activity.

## Data availability

The data supporting this study are available in the article, the supplemental data, or available from the corresponding author upon reasonable request.

## Supplemental data

This article contains supplemental data.

## Conflict of interest

The authors declare that they have no known competing financial interests or personal relationships that could have appeared to influence the work reported in this paper.
