# Supplementary material for: Sensitive UHPLC-MS/MS quantification method for 4β- and 4α-hydroxycholesterol in plasma for accurate CYP3A phenotyping
Source: J Lipid Res. 2022 Feb 16;63(3):100184. doi: 10.1016/j.jlr.2022.100184 (PMC8953653; doi:10.1016/j.jlr.2022.100184)
Supplement: Supplemental Figures S1 and S2 [file mmc1.docx]

**Supplementary Materials**

**Figure S1.** MRM transitions with chemical structures and fragmentation for di-picolinyl ester of 4β-OHC, 4α-OHC and 4β-OHC-D_7_.

**Figure S2.** Chromatogram showing separation of 4β-OHC and 4α-OHC from the isomers comprising 7α-OHC, 7β-OHC, 22(R)-OHC, 22(S)-OHC, 24(S)-OHC, 25-OHC and 27-OHC.
